# Oral health of adolescents in West Africa: prioritizing its social determinants

**DOI:** 10.1186/s41256-023-00313-2

**Published:** 2023-07-19

**Authors:** Deborah Oluwaseun Shomuyiwa, Gemma Bridge

**Affiliations:** 1 Faculty of Pharmacy, University of Lagos, Lagos, Nigeria; 2 School of Earth and Environment, University of Leeds, Leeds, UK

**Keywords:** Social determinants of health, Oral health, Adolescents, Dental caries, Periodontitis, West Africa

## Abstract

Oral health is a major public health issue in West Africa, yet it has gotten little attention. Individual and group disparities in health status are influenced by social determinants of health (SDH), which also affect oral health. Adolescence is a significant transition into adulthood, a time when the SDH can contribute to lifelong health status. This article explored the SDH associated with oral health behaviour, perception, and oral health development amongst adolescents in West Africa. This article engaged articles published in peer-reviewed journals relating to adolescents' oral health and West Africa. The authors undertook this desk review to determine the social determinants of adolescents' oral health in West Africa. The literacy levels and oral health awareness of adolescents, family and social circle influences, socioeconomic status, nutritional levels, and cultural and environmental factors have been identified as important social determinants. Adequate policy implementation with the integration of oral health in schools' curriculum, health systems reorientation with the adoption of oral health delivery in primary health care and expansion in oral health research with the assessment of cultural influences on oral health development have been recommended as interventions to reduce oral health inequalities in West Africa.

## Introduction

Oral health is integral to healthy living and vital to one's quality of life, affecting physical and emotional wellbeing, appearance, and interpersonal relationships [1]. The World Health Organization (WHO) defined oral health as “a state of being free from the mouth or facial pain, oral or throat cancer, oral infection and sores periodontal (gum disease), tooth decay, tooth loss and other disorders that limit an individual’s capacity in biting, chewing, smiling, speaking, and psychosocial wellbeing” [2] According to FDI World Dental Federation (FDI), “oral health includes the ability to speak, smile, smell, taste, touch, chew, swallow and convey a range of emotions through facial expressions with confidence and without pain, discomfort, and disease of the craniofacial complex (head, face, and oral cavity)” [3].

Oral diseases continue to impose a significant global burden, particularly among underprivileged groups, as evidenced by the 2019 report [4]. Oral diseases are estimated to be the most prevalent non-communicable diseases worldwide, affecting nearly 3.5 billion people in 2019 [4]. Dental caries is the most prevalent oral and non-communicable disease (NCD) globally, with 2.3 billion people suffering from caries of permanent teeth and more than 530 million children suffering from caries of primary teeth [5]. Oral diseases are the fourth most expensive disease to treat globally [6], with dental treatment costs averaging 5% of total health expenditure in high-income countries and up to 20% of out-of-pocket health expenditures [7]. Dental caries costs an estimated 442 billion USD annually globally, with 298 billion in treatment costs and 144 billion on lost working days [8]. Stark inequalities exist in oral care provisions as three out of four people affected by oral diseases now live in low-income and middle-income countries [9]. Oral diseases share common risk factors with other non-communicable diseases and can result in similar inequalities. Oral diseases are chronic, progressive, and cumulative. Global health response to oral health development requires the consideration of essential age groups such as adolescents. This perspective aims to study the social determinants of oral health and oral health development of West African adolescents. This will provide a context for modifiable influences of oral health among adolescents in West Africa and set the pace for health systems and policy development for oral health development.

## Oral health of adolescents in West Africa

Adolescence is a significant stage marked by rapid biological, emotional, and social evolution and development. Adolescent age and stage (early, middle and late) and transition to adulthood impact diagnostic, preventive, and restorative treatment decisions and are critical for a human's periodontal status, as periodontal disease causes irreparable tissue damage in late adolescence [10]. The adolescent stage, generally between 10 and 19 years, represents a critical opportunity to influence adult oral health.

**Fig. 1:**
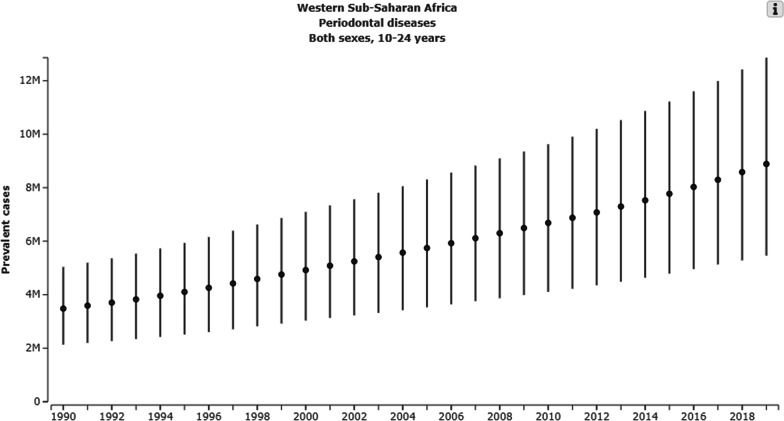
The *Increasing prevalence of Periodontal disease in West Sub-Saharan Africa.* Global Burden of Disease Collaborative Network. Global Burden of Disease Study 2019 (GBD 2019). Seattle: Institute of Health Metrics and Evaluation (IHME); 2020. Available from http://ghdx.healthdata.org/gbd-results-tool

Oral diseases are significant public health problems in West African Adolescents. Adolescents in West Africa live with high unmet dental needs [4]. Cases of oral diseases in adolescents in West Africa have grown in the last 30 years (Fig. 1). Over 2.7 million West African adolescents aged 15 and 19 presented with oral disorders in 2019 [4]. Nigeria represents the majority in the prevalence and incidence of oral diseases in West African adolescents. Dental erosion is common globally among adolescents, while fluorosis is especially prevalent in East and West African countries [11]. Vast unmet dental needs have been identified in adolescents in Sierra Leone, mainly attributed to the minimal dental care provision in the country [12].

The development of dental caries in the permanent teeth peaks in adolescence, a socially and psychologically vulnerable life stage, and is laden with potentially long-term impacts [13, 14]. Poor oral health is associated with pain or discomfort, tooth loss, reduced oral functioning, deformity, missed school time, and mortality from oral cancer, so adolescents with poor oral health can have a worsened quality of life and poorer life outcomes [15]. In Nigeria, 15–58% of adolescents above 15 years present with periodontal diseases, which significantly affect the quality of life [16]. Some reports indicate that the frequency of dental caries in Nigeria is as high as 80% among adolescents compared to almost 35% in the UK [16, 17]. In Ghana, prevalent oral conditions among adolescents were caries, periodontal disease, and malocclusion [18]. In Burkina Faso, Mali, Niger, Nigeria, and Senegal, Noma, a debilitating gangrenous disease of the orofacial region, is particularly prevalent [19].

The West African adolescent population are disproportionately affected by these disorders. These oral health disparities can be found in the distribution of oral health services, accessibility, utilization, treatment outcomes, oral health knowledge and practices, insurance coverage, oral health-related quality of life, and the prevalence of oral diseases, to name a few [20].

## Social determinants and oral health

According to the World Health Organization, the social determinants of health (SDH) are non-medical factors that influence health outcomes [21]. Social determinants are essential as they account for about 30–55% of health outcomes globally [22]. Social determinants of health are widespread, affecting a broad spectrum of oral health outcomes and risk factor exposure. These factors, known as modifiable risk factors, are shared with major non-communicable diseases such as diabetes, cancers, and cardiovascular diseases [23]. SDH are shaped/influenced by expansive sets of forces and are highly complex, encompassing a range of issues. Biological factors like age, sex, and hereditary factors are intrinsically unmodifiable. Social determinants are factors modifiable by behavioural or lifestyle changes [21]. Social determinants of oral health are a range of external factors, including individual behaviour, lifestyle choices, social gradient, and systemic inequalities [23]. The social determinants that may impact the oral health outcomes of adolescents in West Africa include oral health literacy, socioeconomic status, urban–rural differences, health systems, early childhood nutrition, environmental factors, and social and cultural conditions (Table 1).

**Table 1:** Social Determinants that affect oral health outcomes of Adolescents in West Africa

Social determinants	Presentation in West African countries	Impact on adolescent oral health
Oral health literacy	Low literacy rates in sub-Saharan African countries, such as Burkina Faso, Chad, Gambia, Guinea Bissau, Mali, Niger, and Senegal, below 50%, impacting the ability to read and engage with health information.	*Low education and literacy levels can result in the following poor health behaviors*: Infrequent tooth brushing. Non-use of fluoridated toothpaste. Low dental service utilization. Frequent consumption of refined carbohydrates. Cigarette smoking.
Socioeconomic status	Low income in West African families, with all but four countries in West Africa listed as low-income countries, impacts access to oral health-promoting materials like toothbrushes and fluoridated toothpaste.	Socioeconomic status affects oral hygiene practices. Adolescents in resource-limited settings are more affected by chronic oral diseases. Low-income families have less access to essential oral health services and may seek alternative treatments.
Urban–rural differences	Oral diseases are most significant in rural and poor areas of West Africa, with people in rural locations being more likely to be poorer, less health-aware, have more caries, have fewer teeth, lack health insurance, and have less money to spend on dental treatment.	Rural and poor populations experience higher rates of oral diseases and Limited access to dental care, health insurance, and education. These disparities are also linked to lesser health literacy and poor utilization of medical services.
Healthcare systems	The existing gaps in the healthcare system in West African countries influence access to oral healthcare, with structures, workforce training, laws, regulations, and accepted practices impacting individuals' and populations' oral health.	Complexities in accessing quality and affordable oral healthcare. Health-seeking behaviour for oral diseases are unplanned and ad hoc. Regular teeth cleaning and dental visits less common among rural adolescents.
Early childhood nutrition	Unhealthy food choices, including high free sugar intake for children and adolescents and undernutrition/nutritional deficiencies in Western Sub-Saharan Africa, impact oral health by increasing the risk of dental caries and gum diseases and affecting dentition development.	Excessive consumption of sweets and beverages leads to dental decay even in high social classes. Compromised nutrition impacts oral development and contributes to oral diseases in the long term.
Environmental factors	Access to piped water in urban regions, especially in Ghana, Mali, and Senegal, is a significant determinant of oral health. Physical environmental factors such as exposure to air pollution and access to oral health-detrimental substances, like recreational drugs, can impact oral health behaviour.	Periodontal decay and oral deterioration can impact oral health outcomes, extending into adulthood.
Social support	Family, ethnicity, and childhood experiences influence oral health beliefs.	Promoting oral care behaviours in children through observed practices and attitudes improves their oral health. It establishes lasting oral hygiene habits into adulthood. Adverse childhood experiences such as physical and mental abuse and bullying can diminish self-esteem and increase the likelihood of poor oral health.
Culture	Chewing sticks for cleaning and traditional medicine as the first call for oral services is prevalent.	Traditional medicine, is affordable, socially and culturally acceptable, and accessible. It plays a crucial role in relieving acute dental pain in underserved rural areas and performing other practices like tooth extractions.

### Oral health literacy

Oral health literacy refers to the degree to which individuals can obtain, process, understand and utilize basic health information and services necessary to make appropriate oral health decisions [24]. Low levels of education and literacy across West Africa are risk factors for adolescent caries as countries like Burkina Faso, Chad, Gambia, Guinea Bissau, Mali, Niger, and Senegal are among the least literate African countries with literacy rates below 50% [25]. Low literacy levels impact the ability to read and engage with health information, leading to poor health perception and behaviours [26]. This will include low frequency of tooth brushing, non-use of fluoridated toothpaste, poor dental service utilization, high frequency of consumption of refined carbohydrates, and cigarette smoking in some settings.

Parents' educational background and literacy levels strongly correlate to child development [27]. Poor oral health education and limited access to care are characteristic of vulnerable and orphaned adolescents [28]. Dental professionals primarily imparted oral health education, mainly in health provision settings. Topics like tooth decay, oral hygiene, and nutrition for oral health in school environments have a long-term influence on oral health knowledge, perception, and status [29]. School-based strategies for oral health, which are very few in West African schools, have been identified as effective in improving oral health behaviour [30].

### Socioeconomic status

Socioeconomic status, a measure of social standing and economic resources, often reveals inequities in accessing and utilizing resources and healthcare services. Adolescents' socioeconomic status can be assessed according to family income, accommodation, demography, and parents' occupation and educational level [31]. With a combination of family members' social and economic status, there is a stable and robust correlation between socioeconomic status and the health and wellbeing of children [16]. This relationship persists from childhood into adolescence and is vital to overall wellbeing [32].

For family income, the association between low income and poor health has been seen since early childhood [31]. Regional affluence is related to access to essential services, as evidenced by significant gaps between the rich and the less affluent regarding access to essential services like healthcare [33]. Socioeconomic status also significantly influences the type and quality of dental services groups seek. Oyedele demonstrated that children with low socioeconomic status were significantly less likely to accept the caries-related preventive and curative services offered [22]. While the affluent sought dental treatment in private dental clinics, the disadvantaged groups used traditional healers, home treatments, patent medicine dealers, and more [34].

Socioeconomic status is also directly related to oral hygiene practices. Adolescents living in resource-limited settings are more likely to be significantly affected by chronic oral diseases [10]. This can be asserted by the fact that these high-income families can successfully buffer adolescents from the adverse impact of oral conditions [31]. These parents are successfully able to manage their children's chronic conditions. Many West Africans emerge from low-income families, with all but four countries in West Africa listed as low-income countries [35], most of whom live in rural regions. The availability of oral health-promoting materials like toothbrushes and fluoridated toothpaste for tooth cleaning are less in families with low income [36].

### Urban–rural differences

Globally, inequalities persist between urban and rural communities. These disparities are seen in several areas, including the distribution of oral health services, accessibility, utilization, treatment outcomes, oral health knowledge and practices, health insurance coverage, oral health-related quality of life, and prevalence of oral diseases [20]. Reflecting these disparities, oral diseases in West Africa are most significant in the rural and poor. People who live in rural locations are more likely to be poorer, less health-aware, have more caries, have fewer teeth, lack health insurance, and have less money to spend on dental treatment. Low levels of education, which are frequently prevalent in rural locations, have been linked to lesser health literacy and poor utilization of medical services. The odds of presenting dental caries in Sierra Leone between the ages of 12 and 15 years were significantly higher in rural areas [12].

### Healthcare systems

The existing healthcare system also influences access to healthcare. Oral health systems are an interplay of structures, workforce training, laws, regulations, and accepted practices to improve individuals' and populations' oral health. Improving access to oral health care is a critical first step to improving oral health outcomes and reducing disparities. Access to quality health care facilitates the maintenance of good oral health and promotes early diagnosis and prompt treatment of diseases. Oral disparities are fostered by the complexities in accessing effective and quality oral healthcare. Widespread poverty in the region has ensured that health-seeking behaviour for oral diseases is unplanned and ad hoc [32]. Dental caries are more prevalent among rural adolescents, as urban adolescents have better access to regular teeth cleaning and dental visits. Toothpaste and toothbrushes for tooth cleaning are more common among urban populations than rural populations in Nigeria's Port Harcourt, and Lagos states [37].

Promoting oral health and clinical management requires a dedicated health workforce equipped with knowledge, skills and competencies and facilities to integrate the performance of their tasks. Western sub-Saharan Africa represents the lowest absolute numbers and professional-to-population ratios globally in the oral health workforce [14]. The oral health of populations is not a high priority in West African nations, as in Nigeria [38]. This poor utilization of oral care services can be associated with inequity in the distribution of oral health personnel, discrepancies in health insurance coverage, and high financial commitment to access dental care.

The geographic isolation of rural areas and workforce shortages are predominant, and where there is some provision for dental health, the scope of services is minimal. In many West African countries, screening services provision is absent as many patients only present for dental care because of persistent or prominent dental pain or its associated symptoms [34]. The African healthcare environment is known widely for the skewed healthcare environment beset with fear, increased uncertainties, and poor service offerings [35]. Geographically 'poor' regions are known for neglect and the lack of coordinated and dedicated healthcare systems, including oral health [16]. The underserved and vulnerable communities are deprived of essential care. Systemic inequities are prevalent in these communities, and this crucial developmental influence is taken up to adulthood from adolescence.

### Early childhood nutrition

Nutrition and oral health are closely related. Changing food patterns have been indicated to influence oral health development globally. Socioeconomic development, urbanization and globalization have drastically changed food production, preparation, and consumption [40]. Economic instability has been identified to negatively impact food and beverage choices resulting in unhealthy food choices for growing children and adolescents [6]. However, poor oral health outcomes due to poor food choices can also affect children living in high-income areas in West Africa [39]. Globally, an unhealthy diet, especially one high in free sugar, is a crucial risk factor for oral degradation [23]. Consumption of these free sugars—notably present in manufactured food- can be detrimental to oral health development as they cause dental decay in children and adolescents [40]. Undernutrition and nutritional deficiencies also represent a public health challenge in Western Sub-Saharan Africa [4]. Compromised nutrition influences oral development leading in the long term to oral diseases, including edentulousness [41]. Optimal nutrition is required for optimal calcification, development, and growth in the primary and permanent dentition as serious. Inappropriate interactions between microorganisms and a malnourished or immunocompromised host can lead to serious oral health problems [42].

Dental decay due to oral degradation is also highly prevalent in the high social class. The availability of sweets and beverages in homes can facilitate excessive consumption and, thus, dental decay [43]. The prevalence of early childhood caries and tooth loss in Mali's urban children is linked to the considerably higher consumption of sweets in urban regions compared to rural areas [44]. Media and sugar marketing can influence parents and caregivers when feeding their children sweets and snacks [20]. Parents asserted that advertising and marketing on television attract adolescents, and their affordability and availability foster excessive consumption [43].

### Environmental factors

Environmental influences, which relate to interactions between people and the environment that influence diseases, injury, and oral disability, are essential in oral development. In Ghana, Mali, and Senegal, urban regions had higher access to piped water in the home [16]. Dental caries, fluorosis and their association with fluoridated water are the most frequently studied environmentally determined oral health conditions [45]. Fluoridation has a long history of safety and high efficacy in preventing and controlling caries, with benefits extending into adulthood [10]. Insufficient bioavailability of fluoride from drinking water and other sources can impair dental status. The need for supplementary fluoride intake has been identified in the Gambia as natural dietary fluoride availability is low [46].

Physical environmental factors such as exposure and proximity to air pollution and access to oral health-detrimental substances such as recreational drugs influence oral health behaviour [45]. Tobacco use is implicated as the cause of 50% of all periodontal diseases and is an important risk for periodontal decay [23]. Peer smoking and family conditions such as polygamy, smoking parents, and divorced or separated parents had the most substantial influence on adolescents smoking lifestyle [47], contributing significantly to oral deterioration.

### Social support

Oral health outcomes of populations in West Africa are influenced by oral health systems and inherent societal value systems [27]. Family, ethnicity, and race are essential variables of the oral health behaviour of adolescents [31]. The family is an essential social support system that can influence children and adolescents' oral health and behaviour. Parents' and entire families' oral habits and lifestyles influence oral health behaviour [27]. Maternal attitude to oral health significantly predicts children's oral health, and mothers are crucial decision-makers regarding family oral healthcare in Nigeria [48]. Parents' and Parents'/caregivers' knowledge of preventive measures, attitudes, effective treatments, oral care, and hygiene practices is imparted to the children or wards, usually by observational measures. These practices, attitudes and interventions promote oral care behaviours in children, improving their oral health and developing lasting oral hygiene behaviours extending into adulthood.

Adverse childhood experiences, physical and mental abuse and bullying, increase the likelihood of poor oral health as it emaciates self-esteem. With literature on the relationship between a child's adverse social experiences and oral health for African populations scarce, the importance of culture and the social setting in moderating behaviour and managing these social stresses deserves notice. Social support, peers inclusive, can serve as a buffer against stresses and direct a more stable and adaptive physical and emotional development, especially in oral health, as in Nigeria, where caries prevalence is lower in children and adolescents who receive social support with lower self-esteem [49].

### Culture

West Africa is socially and culturally diverse, with powerful religious beliefs influenced by its rich history, identity, and culture [50]. Moving from availability and access to oral health services, socio-behavioural and cultural factors have been identified as important factors [51]. Cultural nuances in dietary practices are guided by seasonality, shared belief and tradition. Traditional medicine is accessible, affordable, and socially and culturally acceptable. They are essential in relieving acute dental pain in underserved rural areas and also conduct other practices such as tooth extractions [52]. In the majorly urban Kadiogo province of Burkina Faso, the local populace relies on plant products when dealing with a broad range of oral concerns [53].

Some populace in the urban regions utilizes both modern medicine and traditional healers [52]. Chewing sticks for cleaning and strengthening teeth is a well-known tradition in West Africa and most of Africa. Muslims prefer to use chewing sticks as it is said to have been supported by Mohammad for maintaining healthy teeth and gums and offers adequate safety [54]. Oral health development has intricate relations with inherent culture.

### Recommendations

#### Policy development

Social determinants are essential, especially in adopting holistic approaches to improve oral health, and they influence oral health development, access to care, and oral habits and behaviours. Oral health policies must be developed to emphasize the roles of social determinants in health development. Promoting oral health amongst adolescents in West Africa requires strategic and well-aligned interventions involving integrated oral health development policies. These policy actions should improve daily living conditions and reduce the decline of the social gradient. Health promotion interventions should also enable the development of age-specific school oral health programs and training, creating an oral-health enabling environment, revamped oral health promotion and advocacy and integration of oral health into the primary health care service. School nutrition policies will also be integral to improving the foods and beverages available in schools.

The policy framework should facilitate the integration of oral health in primary healthcare development in West Africa, sustainable school oral health programs deployment and multisector actions involving ministries and non-governmental organizations (NGOs). The contribution of health sector partners to oral health across the West Africa region should include the development of context and regional-based standard practices, baselines and guidelines for oral health monitoring, evaluation and management. This is important for compliance and sustainability of interventions and practices. Improved access is also attainable as coordinated, and dedicated attention to oral health or accessing health care must be implemented.

#### Integration of oral health in primary healthcare

Over the years, the African Region has developed a network of facilities and infrastructure to support primary healthcare delivery, utilizing a system of health posts and primary health centres with community spirit and participation driving the implementation [36]. Incorporating Oral health into the general healthcare system, especially the primary healthcare structure, with sustained political will and adequate funding, will optimize access and utilization. Its design is founded on public healthcare's social acceptability and participation in Africa. Adequate access to oral healthcare services is a significant concern affecting all healthcare aspects in a population's drive to attain universal health coverage.

#### Oral health promotion

Education and sensitization of adolescents in West Africa on optimal oral health are central to oral health promotion. In furtherance of the WHO goal to increase the uptake of preventive oral self-care, there is a need to design adaptable public health programs to ensure that adolescents place high prerogative on oral preventive measures. The school provides a practical setting for oral health promotion and disease prevention. Oral health should be incorporated into the curricula of West African schools to raise awareness among adolescents. School programs should be designed to motivate different age groups to maintain oral hygiene, emphasizing the appropriate technique and frequency of teeth brushing and flossing. Evidence has shown that motivational interviewing is more effective in evoking favourable changes in the oral health patterns of adolescents and preventing dental caries behaviours [55].

These programs should include collaborative measures with parents. While schools, friends, and other social settings are essential in early oral health development, parents play the most critical roles in their children and adolescents [56]. Digital innovations will also exert disruptive pressures on oral health promotion and health delivery. Mobile apps can be integral in facilitating a change in oral hygiene behaviour. Advances in information management and innovations such as telehealth and precision medicine will be integral in bridging the knowledge and access gap. Prioritizing the delivery of dental public health education through mass and social media will improve access to oral health care information.

#### Research on social and cultural influences

There is a paucity of data on the extent and distribution of the effects of several social factors on oral health development. Social, cultural, and traditional oral health practices and perceptions in West Africa should be succinctly identified and studied. Exploring their positive and detrimental effects on oral health development is crucial. Assessing the synergy between cultural acceptance and oral health development and comparing these practices with modern oral hygiene and health maintenance is paramount. Studying these relations is vital in designing and implementing strategies and frameworks for future preventive and treatment programs. High levels of cultural competence will ensure sustainable local development. Programs with particular consideration for inherent social and cultural values can improve the acceptance and utilization of preventive services and quell oral health disparities. The pluralistic nature of the African health systems suggests that the systematic integration of traditional therapeutic methods into primary oral health care delivery can hold significant value. Research on safe and improved remedies for oral diseases is vital for integrating traditional medicines into primary oral health care.

## Conclusions

Oral diseases are global public health concerns. Social determinants have accumulative effects on health and health trajectories transferred across generations. With most oral diseases mostly preventable despite their high prevalence, the international community should focus on reducing inequalities, especially in resource-limited settings like West Africa. Adolescents have potential and are a tremendous resource, but they require adequate skills and, importantly, wholesome health. In this era of healthcare reform, more emphasis on access, utilization, quality of care, and the impact on population health is crucial. Interventions that bring quality oral healthcare services across different populations broaden oral health-related knowledge and reduce the access gaps initiated by inadequate economic resources and social and cultural influences must be implemented and sustained to promote “health for all”.

## Data Availability

Not applicable.

## Abbreviation

SDH: Social determinants of health
